# Association of the Type 2 Diabetes Mellitus Susceptibility Gene, *TCF7L2*, with Schizophrenia in an Arab-Israeli Family Sample

**DOI:** 10.1371/journal.pone.0029228

**Published:** 2012-01-11

**Authors:** Anna Alkelai, Lior Greenbaum, Sara Lupoli, Yoav Kohn, Kyra Sarner-Kanyas, Edna Ben-Asher, Doron Lancet, Fabio Macciardi, Bernard Lerer

**Affiliations:** 1 Biological Psychiatry Laboratory, Dept. of Psychiatry, Hadassah - Hebrew University Medical Center, Jerusalem, Israel; 2 INSPE, Scientific Institute San Raffaele, Milan, Italy; 3 Department of Sciences and Biomedical Technologies, University of Milan, Italy; 4 Department of Molecular Genetics, Weizmann Institute of Science, Rehovot, Israel; 5 Department of Psychiatry and Human Behavior, University of California Irvine, Irvine, United States of America; University of Chicago, United States of America

## Abstract

Many reports in different populations have demonstrated linkage of the 10q24–q26 region to schizophrenia, thus encouraging further analysis of this locus for detection of specific schizophrenia genes. Our group previously reported linkage of the 10q24–q26 region to schizophrenia in a unique, homogeneous sample of Arab-Israeli families with multiple schizophrenia-affected individuals, under a dominant model of inheritance. To further explore this candidate region and identify specific susceptibility variants within it, we performed re-analysis of the 10q24-26 genotype data, taken from our previous genome-wide association study (GWAS) (Alkelai et al, 2011). We analyzed 2089 SNPs in an extended sample of 57 Arab Israeli families (189 genotyped individuals), under the dominant model of inheritance, which best fits this locus according to previously performed MOD score analysis. We found significant association with schizophrenia of the *TCF7L2* gene intronic SNP, rs12573128, (p = 7.01×10^−6^) and of the nearby intergenic SNP, rs1033772, (p = 6.59×10^−6^) which is positioned between *TCF7L2* and *HABP2*. *TCF7L2* is one of the best confirmed susceptibility genes for type 2 diabetes (T2D) among different ethnic groups, has a role in pancreatic beta cell function and may contribute to the comorbidity of schizophrenia and T2D. These preliminary results independently support previous findings regarding a possible role of *TCF7L2* in susceptibility to schizophrenia, and strengthen the importance of integrating linkage analysis models of inheritance while performing association analyses in regions of interest. Further validation studies in additional populations are required.

## Introduction

Chromosome 10q is remarkably rich in linkage findings for schizophrenia and bipolar disorder [1]. Seven reports in different populations have demonstrated linkage of the 10q24–q26 region to schizophrenia [2], [3], [4], [5], [6], [7], [8]. The studies, demonstrating significant and suggestive schizophrenia linkage to 10q24-q26, greatly encourage a search for specific schizophrenia susceptibility genes in this region. Given the difference between studies in localization of linkage peaks and the fact that peaks in this region were not always the best detected ones in the cited studies, it is reasonable that the region may harbor multiple schizophrenia susceptibility genes with differential contributions to the phenotype in terms of variant frequency, effect size and mode of inheritance rather than a single schizophrenia susceptibility gene [9].

Lerer and collaborators (2003) [4] previously performed a genome-wide linkage study of schizophrenia in a unique, homogeneous sample of Arab-Israeli families with multiple schizophrenia affected individuals and found suggestive linkage to schizophrenia of the 10q24–q26 region, spanning from D10S583 (94 Mb) to D10S217 (129 Mb). In a follow-up publication (Alkelai et al, 2009) [1] we further explored this region in exactly the same Arab sample, by genotyping additional markers and applying additional analytic approaches. While calculating the best-fitting penetrance for the 10q24–q26 locus by maximization of parametric LOD scores over genetic model parameters (MOD score analysis by varying penetrances and disease allele frequency), we showed that the 10q24–q26 locus had a dominant mode of inheritance in the studied Arab-Israeli sample. We refined the linkage region to D10S222 (105.3 Mb) - D10S587 (125.2 Mb) and also demonstrated genetic interaction of this locus with an additional locus, 6q23.3, which was significantly linked to schizophrenia [1]. Although the 10q24–q26 region harbors a large number of protein coding genes (∼130), many of them expressed in the CNS, specific schizophrenia susceptibility genes have not been identified in our sample.

Schizophrenia is a multifactorial, polygenic disorder. A large number of genetic variants may be involved in its genetic background, some of them common, and others rare [10]. The inheritance model of schizophrenia is unknown and the correct model probably differs between risk markers. Our group recently performed a genome-wide association study (GWAS) for schizophrenia in an extended sample of Arab-Israeli families incorporating the families from our original report [11]. In the GWAS we used the additive model of inheritance for the analysis of the data, but none of the SNPs in the 10q24–q26 candidate region reached genome-wide significance. The additive model is one of the most common methods to analyze GWAS data when no previous assumption about mode of inheritance is known. However, as explained above, we showed (by MOD score analysis) that the 10q24–q26 locus had a dominant mode of inheritance in the studied Arab-Israeli sample [1]. We raised the hypothesis, that in this region the genetic contribution to schizophrenia should be tested under a dominant model. Therefore, to be consistent with our previous studies, we have chosen the dominant model for re-analysis of the 10q24–q26 region in the current work. We performed an association study of 2089 region positioned SNPs with schizophrenia using the best-fitting dominant model of inheritance, while appropriately correcting for multiple testing.

## Methods

### Ethics Statement

All participants gave written informed consent. The study was approved by the Helsinki Committee (Internal Review Board) of Hadassah – Hebrew University Medical Center, Jerusalem, Israel.

### Sample

The studied sample was drawn from an ethnically homogenous Arab population, recruited at the Taibe Regional Mental Health Center in Israel, and included 58 nuclear families with 198 genotyped individuals of whom 95 are affected [4]. Additional information about the studied sample may be found in ‘Detailed description of the clinical sample and diagnostic methods’ (Information S1). The relatively small sample size in our study is balanced by the unique nature of the population and decreased genetic heterogeneity. Subjects with medical records of hospitalizations and clinic care were questioned for psychiatric symptoms in the family according to the Family History Research Diagnostic Criteria (FH-RDC) [12] and were interviewed with the Schedule for Affective Disorders and Schizophrenia- Lifetime Version (SADS-L) [13] to establish psychiatric diagnosis. Research Diagnostic Criteria (RDC) [14] and the Diagnostic and Statistical Manual of Mental Disorders, Fourth Edition (DSM-IV) [15] were used for establishment of lifetime diagnoses using a best estimate consensus procedure [16].

### Genotyping

The Arab-Israeli sample was genotyped at the Platform of Genomics and Bioinformatics, University of Milan on the HumanCNV-370 BeadArrays (Illumina, San Diego, USA). Normalized bead intensity data obtained for each sample were analyzed with Illumina GenomeStudio 1.0.2 software [17], [18]. PLINK version 1.06 software [19] was used to perform Quality Control (QC) of the data. The procedure included: evaluation of call rate; check of SNPs with (1) no calls, (2) genotyping rate less than 0.9, (3) MAF less than 0.05; Hardy-Weinberg equilibrium (HWE) testing (p<0.00001) in parents; and exclusion of individuals with missing genotyping >10%. We also checked for the assessment of genetic homogeneity according to the family, sex-check and Mendelian transmission rate. SNPs with more than 10% and families with more than 5% Mendelian error rate were discarded. The sex for each subject was estimated by the GenomeStudio software. After QC procedure 57 nuclear families with 189 genotyped individuals and 307472 autosomal SNPs remained available for the association analysis [11].

### Statistical analysis

For statistical analysis, we used PBAT Version 3.6 [20] which is suitable for analysis of samples made up of different family-types. PBAT statistics were calculated under the null hypothesis of “linkage-and-no-association” and using the sandwich option (sw) for robust estimation of the variance, conditioning on traits and parental genotypes. After restricting the number of informative families to ten and QC procedure, 2089 SNPs located in the 10q24–q26 linkage region were available for analysis. As explained in introduction, we used the dominant inheritance model in our transmission disequilibrium test (TDT). Since this data was previously analyzed by the additive model (as part of the GWAS [11]), we calculated Bonferroni correction value for multiple testing in relation to two analysis models per SNP (2×2089 tests). We used PLINK [19] to estimate effect sizes for the implicated loci in a subset of the sample that included only trios (since this option is not available with PBAT).

To calculate power for dominant model, under a family-based association framework, we used the approach suggested by Lange and Laird (2002) [21] for FBAT, using the program PBAT. The risk for schizophrenia according to the genotypes was modeled by implementing a dominant model. We assumed that the marker locus and the disease locus are different, and power was evaluated for different marker allele frequencies ranging from 0.05 to 0.5. Assuming a region-wide significance level, our analysis indicated that the sample has a power of 30%−100% to detect significant association of alleles with frequencies ranging from 0.05 to 0.5 under a transmission disequilibrium test (TDT) design and a dominant model.

## Results

**Figure 1:**
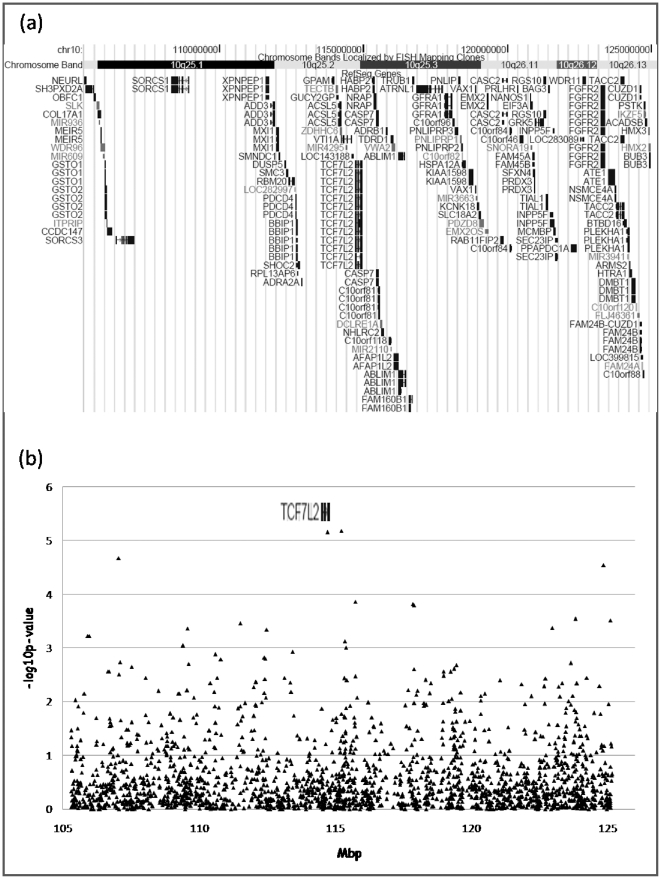
The 10q24-26 region. (a) Graphical representation of the linkage region including the genes within it. The map of the linkage region was adapted from UCSC Genome Browser (http://genome.ucsc.edu/) (Mar. 2006 (NCBI36/hg18) assembly) (b) -log10(p-values) of all the SNPs analyzed in the 10q24-26 region, employing the dominant model and according to the position of the SNPs on the chromosome.

**Figure 2:**
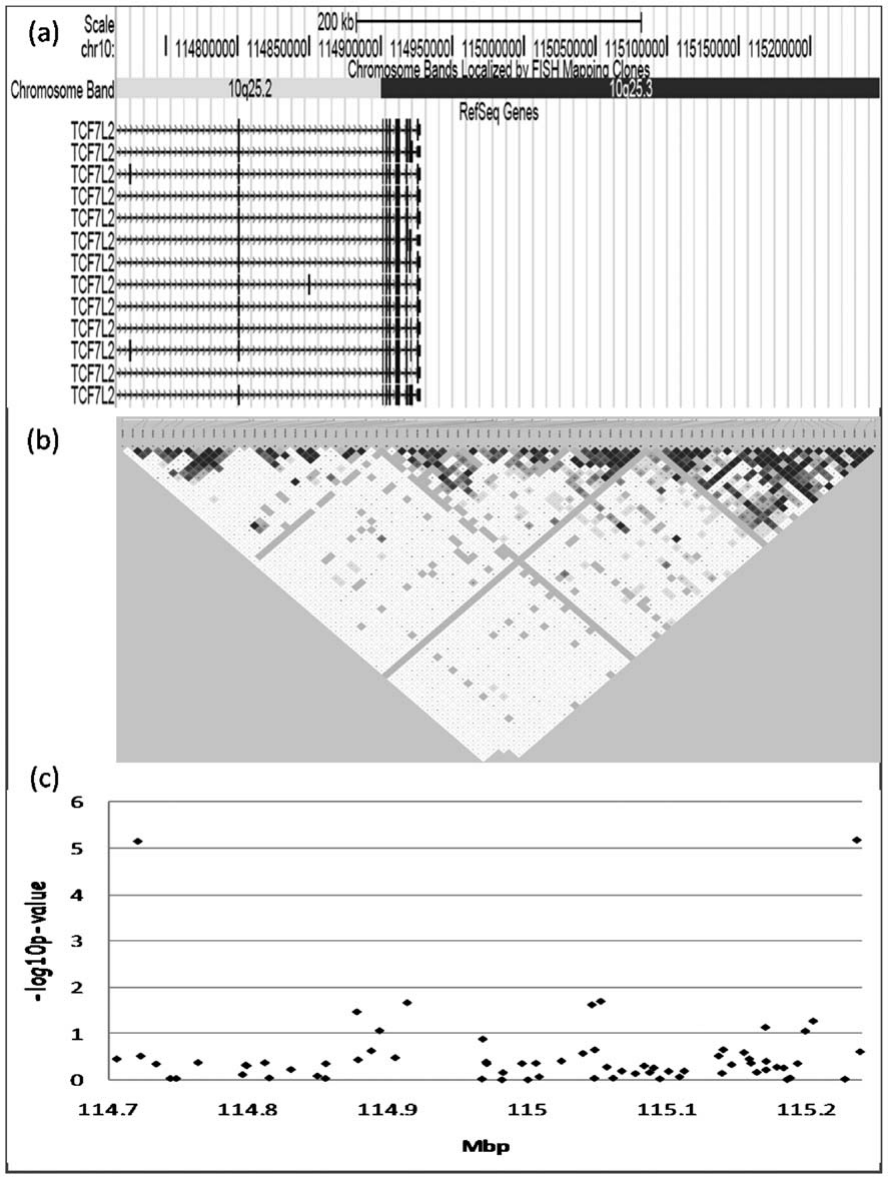
The *TCF7L2* gene region. (a) The map of the genomic region adapted from UCSC Genome Browser (http://genome.ucsc.edu/) (Mar. 2006 (NCBI36/hg18) assembly) (b) Haploview representation of the LD structure between two *TCF7L2* significant SNPs (rs12573128 and rs1033772). (c) -log10(p-values) of all the SNPs analyzed between rs12573128 and rs1033772.

We focused on the 10q24–q26 linkage region and re-analyzed the available genotype data of 2089 SNPs positioned in this area (taken from our previous GWAS (Alkelai et al, 2011) [11]) in order to study association with schizophrenia under the dominant model. We found two significant associations with schizophrenia that survived region-wide correction for multiple testing (2089×2 tests , p-value<1.197×10^−5^), under the dominant model: *TCF7L2* intronic SNP rs12573128 (p = 7.01×10^−6^) and the intergenic SNP, rs1033772 (p = 6.59×10^−6^) (Figure 1, Table 1). rs1033772 is located 318 kb from *TCF7L2* and 65 kb from *HABP2*. The R-square and D′ between these two SNPs are 0 and 0.14 respectively; therefore these two SNPs are not in linkage disequilibrium (LD) (Figure 2). Information regarding nominal association of all SNPs in this region (p<0.001) under the dominant model is supplied in Table S1. The dominant and the additive models are partially correlated to each other, and not independent. Therefore, it is expected that at least partial overlap between the top results of the two models will be observed. Information regarding nominal association of all SNPs in this region (p<0.001) under the additive model previously used in the GWAS (Alkelai et al, 2011, [11]) is supplied in Table S2. Overlapping positive results (p<1×10^−3^) regarding the two models, were found for a number of SNPs located within or near different genes, including *TCF7L2* SNP of interest, as well as for additional SNPs in other genes (*SORCS3*, *NRAP*, *GFRA1*, *TACC2*, and *HMX3*).

**Table 1:** Significant results for association with schizophrenia.

SNP	Bp	Allele	Freq	Gen_rate	Mend_err	HW_parents	OR	p-value	*Gene*
rs1033772	115235922	A	0.49	0.96	0	0.68	0.65	6.59×10^−6^	*TCF7L2-HABP2*
rs12573128	114720787	G	0.17	1	0	0.47	0.35	7.01×10^−6^	*TCF7L2*

P-values, allelic frequencies, and Hardy Weinberg equilibrium were obtained using PBAT. Genotyping rate, Mendelian errors, and OR were obtained using PLINK. Abbreviations: HW = Hardy Weinberg equilibrium; allele = minor allele; freq = allele frequency; Gen_rate = genotyping rate, Mend_err = Mendelian errors.

## Discussion

In the current study, we sought to identify novel schizophrenia susceptibility variants within the 10q24–q26 region. The starting point of this work was the prior Alkelai et al. (2009) linkage study which used MOD score analysis to explore this region. This area was reported by our [1], [4] and other linkage studies [2], [3], [4], [5], [6], [7], [8] to harbor schizophrenia susceptibility genes. Based on previous linkage results supporting the relevance of a dominant model of inheritance to this particular sample [1], [4], we performed a region specific association study of 2089 SNPs covering the area (for which genotype data was available from our previous GWAS study (Alkelai et al, 2011) [11]) in a family based sample of Arab Israeli origin, applying a dominant model of inheritance. We found region-wide significant associations of two SNPs, one of them located within *TCF7L2* intron 4 (rs12573128, p = 7.01×10^−6^) and the other an intergenic SNP (rs1033772, p = 6.59×10^−6^), positioned 318 kb downstream to this gene and 65 kb from the *HABP2* gene. Although both associations withstand region-wide correction for multiple testing (2089×2 tests), the findings should be regarded as preliminary and “hypothesis generating” rather than definitive, and require further confirmation in additional samples and populations.

Recently, several converging studies reported association of different and independent genetic variants within or flanking *TCF7L2* with schizophrenia (data summarized in Table 2). Ben-David and colleagues [22] showed association of the rs17746501 SNP (61 kb upstream to *TCF7L2*) with schizophrenia in a large meta-analysis. An additional nearby intergenic SNP, rs11595716, was associated with schizophrenia in an independent sample from Germany, as reported by Need et al (2009) [23], although not in a dominant model. Hansen and collaborators identified significant association of a different *TCF7L2* intronic variant (rs7903146) with schizophrenia in a Danish sample, and this association was replicated in a large multinational European sample of approximately 4,000 schizophrenia patients and 17,500 controls [24]. Interestingly, this particular SNP was also genotyped and analyzed in our Arab sample, but was not associated with schizophrenia. Therefore, evidence is emerging to support involvement of several independent SNPs within or flanking *TCF7L2* in schizophrenia susceptibility; some of them may be population specific.

**Table 2:** Summary of positive association findings near the *TCF7L2* gene (10q25.2-q25.3).

Study	SNP	Position on chromosome 10	Location	P-value
Need et al. 2009	rs11595716	114633926	intergenic	2×10^−3^
Ben-David et al. 2010	rs17746501	114639457	intergenic	7×10^−3^
Hansen et al. 2011	rs7903146	114748339	intronic	5×10^−3^
Current study	rs12573128	114720787	intronic	7×10^−6^
Current study	rs1033772	115235922	intergenic	7×10^−6^

The intergenic SNP rs1033772 is not in LD with the *TCF7L2* gene or with rs12573128, and it is not clear if it exerts a biological influence on this gene. Moreover, since this SNP is closer to another gene, *HABP2*, we should not ignore the possibility that our 10q24–q26 region harbors more than one schizophrenia susceptibility gene, acting independently: *TCF7L2* and *HABP2*. Moreover, a third susceptibility gene for schizophrenia, *FGFR2*, was found in this genomic area by O'Donovan et al (2009) [25]. HABP2 (hyaluronan binding protein 2), named also FSAP (serine protease FVII activating protein) is an extracellular serine protease that binds hyaluronic acid [26]. It is predominantly produced in the liver, circulates in plasma and could cleave pro-urokinase, coagulation factor VII, and platelet-derived growth factor [27]. Due to the current knowledge of biological role of this gene, *HABP2* seems to us as less attractive potential schizophrenia susceptibility gene, compared to *TCF7L2*, although this may not necessarily be true. Nevertheless since intergenic SNPs may potentially influence expression of nearby genes (located 1 Mb from the transcription start site) [28], [29], [30], we cannot exclude the option that the intergenic SNP rs1033772 may affect *TCF7L2* expression. This option should be tested experimentally.

Several limitations should be bear in mind when interpreting our results. As in previous association studies in psychiatric genetics published in recent years, it is possible that our findings are false positive, particularly given the relatively small sample size. Since the true mode of inheritance of schizophrenia is unknown, and use of MOD score analysis could inflate the type I error, it is possible that approximated model parameters do not reflect the real mode of schizophrenia inheritance and the reported results could be spurious. In addition, the correction for multiple testing applied here is suited for a region-wide correction (required to study the a priori research hypothesis) although genotype data were taken from a much larger pool of 307472 autosomal SNPs (originally analyzed under the additive model of inheritance). In this regard the current analysis should be seen as complementary to the original GWAS study, already published (Alkelai et al, 2011) [11]. Last, we do not present here a replication trial in an independent sample, which could have assist (if positive and supports the association) in addressing this issue.

Nevertheless, these substantial limitations are balanced by several factors, supporting the probability of a true positive. First, our homogeneous family-based design is robust against false-positive associations resulting from population stratification. Second, we used strict correction for multiple testing (Bonferroni, applied to the specific region of interest) in spite of the fact that the existence of linkage disequilibrium (LD) in this area may render it overly conservative. Even if multiple testing correction is taken for all three main possible inheritance models used in the literature in the context of schizophrenia (including the recessive model), the results still pass the correction threshold. Third, the specific inheritance model studied here (dominant) was chosen based on evidence provided from the Arab Israeli families. The dominant model of inheritance is acceptable and widely used in schizophrenia genetics research, both in candidate gene studies (eg. Kim et al, 2010; Xu et al, 2010; Okochi et al, 2009; Kim et al, 2008) [31], [32], [33], [34] and in GWASs (eg. Shifman et al, 2008) [35]. At the molecular biology level, the implication of a *TCF7L2* effect on schizophrenia under the dominant model, is not clear and should be further investigated. Fourth, our data are in line with the results of other groups [22], [23], [24].

It is presumable that the identified intronic association signal is in linkage disequilibrium with a schizophrenia causative variant, which is specific to our particular Arab study sample. The family based sample belongs to an ethnically homogeneous group that has a high birthrate, an unusually high level of consanguinity and a low rate of intermarriage with other population groups [36], [37]; therefore a founder effect may exist in this population. On the other hand, additional reported associations of *TCF7L2* with schizophrenia, described above (Table 2) probably represent more generalized risk variants. Further studies including sequencing, in this specific sample (whole *TCF7L2* sequencing, searching for a point mutations) and in independent samples, are required to properly address the issue of generalizability of the association to other populations. In this regard, we are encouraged by our previous success in verifying results following fine mapping in another linkage region (6q23) in the same unique sample of Arab Israelis. This led to identification of *AHI1* as schizophrenia susceptibility gene [37] which was further replicated in an Icelandic case control sample [38] and recently in a large European and Spanish/German samples [39], [40]. *AHI1* is located in the linkage region which was previously shown to genetically interact with the 10q24–q26 locus in our sample [1].

*TCF7L2* is a confirmed type 2 diabetes (T2D) susceptibility gene and is now a major focus of T2D genetic and molecular research. Following the first report of Grant and colleagues [41] of association of *TCF7L2* gene variants with T2D, a large number of studies in various populations have replicated the original findings [42], [43], [44], [45], [46], [47], [48], [49], [50], [51], [52], [53]. Several meta-analyses further supported this robust finding [54], [55]. Polymorphism within *TCF7L2* was also associated with increased risk for T2D among schizophrenia patients, in whom the disease is relatively prevalent, as discussed below [56]. The functional role of this gene in humans is under intense investigation [57], [58].

Increased attention is now being given to a possible genetic basis for comorbidity of T2D and schizophrenia [59]. The risk of T2D is higher among schizophrenia patients than in the general population of the same age group, mainly among young males [60], [61]. This is true even if schizophrenia patients are drug naïve [62]. First episode, drug naïve schizophrenia patients had higher fasting plasma glucose levels than controls [63] and a higher incidence of T2D [64]. Nevertheless, it is difficult to determine if diabetes and other glucose metabolism abnormalities stem from schizophrenia itself or from treatment with antipsychotic medication [65]. Lin and Schuldiner [59] proposed that the co-occurrence of the two disorders may be explained, at least partially, by shared genetic risk variants. *TCF7L2* may contain independent variants for both disorders, or variants that exert pleiotropic effect (the same variant causes the two different pathological conditions).

Although much attention had been given to TCF7L2 function in diabetes related organs such as pancreas, adipocytes and intestine, its role in the brain is largely unknown. Lee and collaborators reported high *TCF7L2* expression in thalamic and tectal adult mouse brain structures, with lower expression level in the hypothalamus and additional areas [66]. Further studies showed that *TCF7L2* expression in the CNS is characterized by a variety of splice variants. Nazwar et al [67] detected a differentiation in *TCF7L2* splice variant expression among post-mitotic neurons, immature neural precursors and intestinal epithelia in a murine model. At the primate level, *TCF7L2* is expressed in excitatory neurons in adult male rhesus monkeys [68]. In humans, a unique splice variant was found in the brain, islet and gut and therefore named the “neuroendocrine form”. This splice variant is highly expressed in the thalamus, occipital lobe and hypothalamus [69]. Functionally, TCF7L2 is a transcription factor involved in the Wnt/beta-catenin signaling [70]. The Wnt signaling pathway plays role in the CNS development [71], [72], and has been also associated with schizophrenia in a number of studies [73].

In conclusion, we performed re-analysis of the 10q24-q26 region association with schizophrenia. The results demonstrated region-wide significant association of the T2D susceptibility gene, *TCF7L2* with the disease. This report strengthens the importance of integrating several model of inheritance when analyzing association studies in regions of interest. Validation studies in other samples are warranted.

## Supporting Information

Table S1 **The top results for association with schizophrenia (p<1×10^−3^) in the 10q24-26 region, by using the dominant model.** P-values, allelic frequencies and Hardy Weinberg equilibrium were obtained using PBAT. Abbreviations: HW = Hardy Weinberg equilibrium; Allele = minor allele; Freq = minor allele frequency.
(DOC)

Table S2 **The top results for association with schizophrenia (p<1×10^−3^) in the 10q24-26 region, by using the additive model (adapted from Alkelai et al, 2011** [**11**] **).** P-values were obtained using PBAT. The overlapping best results (p<1×10^−3^) with the dominant model are represented in bold.
(DOC)

Information S1 **Detailed description of clinical sample and diagnostic methods.**
(DOC)
