# Supplementary material for: Association of the Type 2 Diabetes Mellitus Susceptibility Gene, TCF7L2, with Schizophrenia in an Arab-Israeli Family Sample
Source: PLoS One. 2012 Jan 11;7(1):e29228. doi: 10.1371/journal.pone.0029228 (PMC3256145; doi:10.1371/journal.pone.0029228)
Supplement: Table S1 — The top results for association with schizophrenia (p<1×10^−3^) in the 10q24-26 region, by using the dominant model. P-values, allelic frequencies and Hardy Weinberg equilibrium were obtained using PBAT. Abbreviations: HW = Hardy Weinberg equilibrium; Allele = minor allele; Freq = minor allele frequency. (DOC) [file pone.0029228.s001.doc]

**Table S1:** The top results for association with schizophrenia (p<1x10^-3^) in the 10q24-26 region, by using the dominant model. P-values, allelic frequencies and Hardy Weinberg equilibrium were obtained using PBAT. Abbreviations: HW=Hardy Weinberg equilibrium; Allele=minor allele; Freq= minor allele frequency.

| **SNP** | **Bp** | **Allele** | **Freq** | **HW_parents** | **Main affect** | **p-value** | **Gene** |
| --- | --- | --- | --- | --- | --- | --- | --- |
| rs1033772 | 115235922 | A | 0.489 | 0.683 | - | 6.60 x10^-6^ | *TCF7L2-HABP2* |
| rs12573128 | 114720787 | G | 0.17 | 0.473 | - | 7.01 x10^-6^ | *TCF7L2* |
| rs7903001 | 107055423 | G | 0.062 | 0.968 | - | 2.12 x10^-5^ | *SORCS3-SORCS1* |
| rs4980259 | 124862504 | C | 0.428 | 0.401 | + | 2.86 x10^-5^ | *ACADSB-HMX3* |
| rs82625 | 115746120 | A | 0.12 | 0.518 | + | 1.37 x10^-4^ | *NHLRC2-ADRB1* |
| rs17094083 | 117850841 | G | 0.067 | 0.965 | - | 1.53 x10^-4^ | *GFRA1* |
| rs2286732 | 117890681 | G | 0.242 | 0.962 | - | 1.60 x10^-4^ | *GFRA1* |
| rs2461224 | 123829539 | A | 0.328 | 0.516 | - | 2.87 x10^-4^ | *TACC2* |
| rs10794614 | 125110144 | G | 0.381 | 0.99 | - | 3.07 x10^-4^ | *CR607950* |
| rs7091635 | 111527585 | G | 0.406 | 0.68 | - | 3.50 x10^-4^ | *SORCS1-XPNPEP1* |
| rs4500422 | 122980137 | A | 0.354 | 0.995 | - | 4.20 x10^-4^ | *WDR11-FGFR2* |
| rs2265217 | 109576563 | C | 0.325 | 0.179 | + | 4.32 x10^-4^ | *SORCS1-XPNPEP1* |
| rs1934930 | 112489238 | G | 0.423 | 0.893 | + | 4.53 x10^-4^ | *RBM20* |
| rs642743 | 105923407 | C | 0.49 | 0.993 | + | 5.97 x10^-4^ | *C10orf79* |
| rs581686 | 105973346 | G | 0.49 | 0.993 | + | 5.97 x10^-4^ | *C10orf79* |
| rs1361853 | 115363267 | A | 0.201 | 0.513 | + | 7.49 x10^-4^ | *NRAP* |
| rs11193593 | 109418366 | A | 0.196 | 0.028 | - | 9.07 x10^-4^ | *SORCS1-XPNPEP1* |
| rs2275799 | 115399830 | A | 0.389 | 0.109 | + | 9.90 x10^-4^ | *NRAP* |
