# Supplementary material for: Association of the Type 2 Diabetes Mellitus Susceptibility Gene, TCF7L2, with Schizophrenia in an Arab-Israeli Family Sample
Source: PLoS One. 2012 Jan 11;7(1):e29228. doi: 10.1371/journal.pone.0029228 (PMC3256145; doi:10.1371/journal.pone.0029228)
Supplement: Table S2 — The top results for association with schizophrenia (p<1×10^−3^) in the 10q24-26 region, by using the additive model (adapted from Alkelai et al, 2011 [11] ). P-values were obtained using PBAT. The overlapping best results (p<1×10^−3^) with the dominant model are represented in bold. (DOC) [file pone.0029228.s002.doc]

**Table S2:** The top results for association with schizophrenia (p<1x10^-3^) in the 10q24-26 region, by using the additive model (adapted from Alkelai et al, 2011 [1]). P-values were obtained using PBAT. The overlapping best results (p<1x10^-3^) with the dominant model are represented in bold.

| **Chr** | **SNP** | **Bp** | **p-value (add)** | **Position** | **Closest gene** | **Bp from the closest gene** |
| --- | --- | --- | --- | --- | --- | --- |
| 10 | rs12772784 | 105456798 | 7.59x10^-4^ | intronic | *SH3PXD2A* | 0 |
| **10** | **rs7903001** | **107055423** | **3.73x10^-4^** | **3' region** | ***SORCS3*** | **40440** |
| 10 | rs1336979 | 108942503 | 6.10x10^-4^ | 5' region | *SORCS1* | 28047 |
| 10 | rs12779243 | 108943137 | 6.10 x10^-4^ | 5' region | *SORCS1* | 28681 |
| 10 | rs4918292 | 109035829 | 9.63 x10^-4^ | intergenic | *SORCS1* | 121373 |
| 10 | rs1572711 | 109056842 | 3.20 x10^-4^ | intergenic | *SORCS1* | 142386 |
| 10 | rs921348 | 111697488 | 5.56 x10^-4^ | intronic | *LOC100505933* | 0 |
| 10 | rs10749041 | 112408768 | 9.32 x10^-4^ | intronic | *RBM20* | 0 |
| 10 | **rs12573128** | **114720787** | **1.97** **x10^-4^** | **intronic** | ***TCF7L2*** | **0** |
| 10 | rs290481 | 114913815 | 3.01 x10^-4^ | intronic | *TCF7L2* | 0 |
| **10** | **rs1361853** | **115363267** | **2.33** **x10^-4^** | **intronic** | ***NRAP*** | **0** |
| **10** | **rs17094083** | **117850841** | **7.73** **x10^-5^** | **intronic** | ***GFRA1*** | **0** |
| **10** | **rs2286732** | **117890681** | **4.19 x10^-5^** | **intronic** | ***GFRA1*** | **0** |
| 10 | rs2074986 | 118018604 | 1.84 x10^-4^ | intronic | *GFRA1* | 0 |
| 10 | rs703393 | 119371376 | 5.93 x10^-4^ | 3' region | *EMX2* | 72329 |
| 10 | rs855983 | 119403172 | 9.68 x10^-4^ | intergenic | *EMX2* | 104125 |
| **10** | **rs2461224** | **123829539** | **3.00 x10^-4^** | **intronic** | ***TACC2*** | **0** |
| **10** | **rs4980259** | **124862504** | **1.21 x10^-5^** | **5' region** | ***HMX3*** | **23053** |
| 10 | rs7913911 | 128594603 | 2.83 x10^-4^ | intronic | *DOCK1* | 0 |

1. Alkelai A, Lupoli S, Greenbaum L, Giegling I, Kohn Y, et al. (2011) Identification of new schizophrenia susceptibility loci in an ethnically homogeneous, family-based, Arab Israeli sample. FASEB J 25: 4011-4023.
