# Supplementary material for: Association of the Type 2 Diabetes Mellitus Susceptibility Gene, TCF7L2, with Schizophrenia in an Arab-Israeli Family Sample
Source: PLoS One. 2012 Jan 11;7(1):e29228. doi: 10.1371/journal.pone.0029228 (PMC3256145; doi:10.1371/journal.pone.0029228)
Supplement: Information S1 — Detailed description of clinical sample and diagnostic methods. (DOC) [file pone.0029228.s003.doc]

**Information S1: Detailed description of clinical sample and diagnostic methods**

As described previously [1-4], the Arab Israeli family sample is drawn from an ethnically homogenous population that originated less than 30 generations ago with a limited number of founders and is characterized by an unusually high level of consanguinity, high birth rate and a low rate of intermarriage with other population groups in Israel. Families of Arab Israeli origin with two or more schizophrenia affected members were collected from the Taibe Regional Mental Health Center[3,4]. This clinic serves a population of ~66000 individuals living in adjacent central Israel region Arab villages and towns. All subjects provided written informed consent. The project was approved by the Helsinki Committee (Internal Review Board) of the Hadassah - Hebrew University Medical Center. Including the families recruited to the original genome scan [3], the sample available for the current family based association study is slightly enlarged from previous work [2] and consists of 58 nuclear families (198 genotyped individuals of whom 95 are affected); 36 of the families are "triads" (affected proband plus parents) while the rest include multiple offspring. Four families are multigenerational. Subjects with medical records of hospitalizations and clinic care were questioned for psychiatric symptoms in the family according to the Family History Research Diagnostic Criteria (FH-RDC)[5] and were interviewed with the Schedule for Affective Disorders and Schizophrenia- Lifetime Version (SADS-L) [6] to establish psychiatric diagnosis. Two experienced members of the research team reviewed the completed SADS-L interview form, FH-RDC information and medical records. In cases where consensus was not achieved, the review was done also by the principal investigator. Research Diagnostic Criteria (RDC)[7] and the Diagnostic and Statistical Manual of Mental Disorders, Fourth Edition (DSM-IV)[8] were used for establishment of lifetime diagnoses using a best estimate consensus procedure[9]. Diagnostic evaluations were done without knowledge of the genotyping data. In the previous studies [3,4] broad, core and narrow diagnostic categories were employed. In the present study, we used only the broad category, since this category consistently yielded the strongest results in our previous genome scan and fine mapping studies. In the current sample this category included 71 genotyped subjects affected with schizophrenia according to RDC; 17 genotyped subjects affected with schizoaffective disorder, one genotyped subject affected with unspecified functional psychosis, 3 genotyped subjects had schizotypal features, two genotyped subjects affected with brief psychotic disorder and one genotyped subject with manic disorder. Other potential broad diagnostic category diagnoses are not in fact represented in the sample.

**References**

1. Alkelai A, Kohn Y, Olender T, Sarner-Kanyas K, Rigbi A, Hamdan A, Ben-Asher E, Lancet D, Lerer B (2009) Evidence for an interaction of schizophrenia susceptibility loci on chromosome 6q23.3 and 10q24.33-q26.13 in Arab Israeli families. Am J Med Genet B Neuropsychiatr Genet 150B (7):914-925

2. Amann-Zalcenstein D, Avidan N, Kanyas K, Ebstein RP, Kohn Y, Hamdan A, Ben-Asher E, Karni O, Mujaheed M, Segman RH, Maier W, Macciardi F, Beckmann JS, Lancet D, Lerer B (2006) AHI1, a pivotal neurodevelopmental gene, and C6orf217 are associated with susceptibility to schizophrenia. Eur J Hum Genet 14 (10):1111-1119

3. Lerer B, Segman RH, Hamdan A, Kanyas K, Karni O, Kohn Y, Korner M, Lanktree M, Kaadan M, Turetsky N, Yakir A, Kerem B, Macciardi F (2003) Genome scan of Arab Israeli families maps a schizophrenia susceptibility gene to chromosome 6q23 and supports a locus at chromosome 10q24. Mol Psychiatry 8 (5):488-498

4. Levi A, Kohn Y, Kanyas K, Amann D, Pae CU, Hamdan A, Segman RH, Avidan N, Karni O, Korner M, Jun TY, Beckmann JS, Macciardi F, Lerer B (2005) Fine mapping of a schizophrenia susceptibility locus at chromosome 6q23: increased evidence for linkage and reduced linkage interval. Eur J Hum Genet 13 (6):763-771

5. Andreasen NC, Endicott J, Spitzer RL, Winokur G (1977) The family history method using diagnostic criteria. Reliability and validity. Arch Gen Psychiatry 34 (10):1229-1235

6. Spitzer R EJ (ed) (1977) The schedule for affective disorders and schizophrenia, lifetime

version, 3rd ed. New York State Psychiatric Institute, New York.,

7. Spitzer RL, Endicott J, Robins E (1978) Research diagnostic criteria: rationale and reliability. Arch Gen Psychiatry 35 (6):773-782

8. American_Psychiatric_Association (1994) Diagnostic and Statistical Manual of Mental Disorders 4th Edition. 4th Edition edn. American Psychiatric Association, Washington, DC

9. Baron M, Endicott J, Lerer B, Loth JE, Alexander JR, Simon R, Sharpe L, Gibbon M, Hasin D, Lilliston B, et al. (1994) A pedigree series for mapping disease genes in bipolar affective disorder: sampling, assessment, and analytic considerations. Psychiatr Genet 4 (1):43-55
